# Critical thinking and misinformation vulnerability: Experimental evidence from Colombia

**DOI:** 10.1093/pnasnexus/pgae361

**Published:** 2024-10-15

**Authors:** John A List, Lina M Ramirez, Julia Seither, Jaime Unda, Beatriz H Vallejo

**Affiliations:** Department of Economics, University of Chicago, Chicago, IL 60637, USA; Department of Economics, University of Chicago, Chicago, IL 60637, USA; Department of Economics, Universidad del Rosario, Bogota, Cundinamarca 111711, Colombia; Ethos Behavioral Team, Bogota, Cundinamarca 110111, Colombia; Ethos Behavioral Team, Bogota, Cundinamarca 110111, Colombia

**Keywords:** fake news, misinformation, critical thinking

## Abstract

Misinformation represents a vital threat to the societal fabric of modern economies. While skills interventions to detect misinformation such as de-bunking and prebunking, media literacy, and manipulation resilience have begun to receive increased attention, evidence on de-biasing interventions and their link with misinformation vulnerability is scarce. We explore the demand for misinformation through the lens of augmenting critical thinking in an online framed field experiment during the 2022 Presidential election in Colombia. Data from roughly 2.000 individuals suggest that providing individuals with information about their own biases (obtained through a personality test) has no impact on skepticism towards news. But (additionally) showing participants a de-biasing video seems to enhance critical thinking, causing subjects to more carefully consider the truthfulness of potential misinformation.

**Significance Statement**

The widespread problem of misinformation significantly affects contemporary societies, shaping election outcomes, public health, and social division. Our study investigates how interventions designed to enhance critical thinking can affect individuals’ susceptibility to misinformation. By utilizing experimental data from Colombia, we assess whether encouraging individuals to pause and reflect on their cognitive processes can mitigate their vulnerability to misinformation. Our findings reveal that while highlighting personal behavioral biases alone is ineffective, exposing individuals to debiasing videos appears to promote critical thinking and reduce susceptibility to misinformation.

## Introduction

One would be hard-pressed to find an issue as divisive for modern nations as the spread of misinformation. Yet our understanding of the economic sources of misinformation remains nascent. Given that the spread of misinformation can negatively affect election outcomes (1), health outcomes (2), and increase polarization (3), the foundation of modern societies is critically linked to people’s beliefs and their trust in institutions.

Through an economics lens, much of the work to date addressing misinformation relates to the supply side of the information market, by fact-checking news (4) or using social media initiatives and advertising (5) such as, for example, accuracy prompts. This work has lent important insights into the causes and consequences of misinformation on the supply side. Yet, every market has two sides and to understand the market for misinformation and craft efficient policies to combat its impact, we also need to further our understanding of the demand side.

Our study contributes to the broader social sciences literature that has been growing in the past decade seeking to understand the demand side of misinformation (6). We provide experimental evidence on the effectiveness of debiasing interventions by exploring how nudges to critical thinking skills affect the demand and treatment of misinformation. We leverage the critical thinking framework of (7) to examine if treatments that teach individuals to “slow down” and reflect on their own experiences and behavior makes them less vulnerable to misinformation. To operationalize our approach, we conduct an online framed field experiment to provide evidence relating the vulnerability to misinformation to critical thinking in a context with high probability of fake news prevalence—the Colombian Presidential elections in 2022. Colombia is severely politically polarized (8), and although Colombia’s democracy is one of the longest-standing in Latin America, the country still experiences violence and human rights abuse. Reducing the demand for misinformation may have meaningful impacts on the functioning of democracy.

We conducted an online framed field experiment with 2.235 adults by randomizing individuals into one of four groups. The first group watches a thought-provoking video that shows how automatic thinking and misperceptions can affect our everyday lives. The second group completes a personality test that shows them their cognitive traits and how this makes them prone to behavioral biases. A third group watches both the video and completes the personality test. We benchmark treatment effects against a control group without any intervention. Additionally, we explore four potential mediators in the relationship between our treatments and fake news vulnerability: trust, own discrimination perception, ambiguity aversion, and dehumanization (9–11).^a^

Our main outcomes of interest are skepticism towards news (fake and true, to elicit discernment) and stated willingness to report fake news on social media. Our experimental results show that debiasing subjects by showing them their biases based on evaluations from personality tests on its own has no impact on their skepticism. We furthermore find suggestive evidence that the video interventions are successful in decreasing misinformation vulnerability. Distinguishing between news types, we find that point estimates are larger for neutral than for political news, potentially speaking to a higher stickiness of political misinformation vulnerability. However, individuals in the control group are more likely to consider neutral news reliable than political news suggesting larger treatment impacts of videos on political misinformation. The effect seems to be stronger for fake news targeting the political right. We also find that providing debiasing information in addition to the videos is not more effective than showing the videos on their own—in line with our first result on the ineffectiveness of personality tests to debias individuals.

Overall, these findings provide evidence that exposing individuals to their own behavioral biases is not effective. If anything, we find some evidence that videos that highlight relatable, sympathetic individuals with different backgrounds and beliefs are more effective in making individuals more skeptical. Interestingly, our results suggest that the joint intervention (personality test and video) affects the perceptions about the reliability of true news headlines. However, neither the video intervention nor the personality test alone alters perceptions regarding the reliability of true news headlines overall or covering political news.

This result suggests that while our video intervention seems to fosters critical thinking by cultivating skepticism toward fake headlines and facilitating the differentiation between true and false political news, the tools provided by the video to enhance discernment are limited. When combined with the personality test, individuals generally become more skeptical but struggle to discern between what is genuine and what is fabricated. A promising avenue for future research, following the efforts of (12), lies in identifying tools that not only increase skepticism but also bolster discernment.

While the videos on their own seem to decrease misinformation vulnerability, they are not sufficient to affect the spread of misinformation. Using our measure of reporting fake news tweets we do not find that videos increase tweet reporting. We find, however, that individuals who receive the joint intervention (video and behavioral assessment) are 15% (8.5 pp) more likely to state that they are willing to report the fake nonpolitical tweet (Tweet A). This result suggests that decreasing misinformation vulnerability only leads to (stated) behavior changes if combined with personalized information about own behavioral biases.

Regarding our four potential mediators, we find sizable impacts of our treatments on dehumanization but not on discrimination perception, trust or ambiguity aversion. We find that watching the video decreases by 16% (9.7 pp) and 8% (5.8 pp) the use of negative characteristics to describe the political left and political right, respectively, suggesting a reduction in dehumanization. Again, we find that the personality tests on their own do not affect any of our four indicators and that adding the personality test results to the video does not affect our potential mechanisms differently than with the video alone.

To provide further robustness to our results, we provide estimates under imputation and Lee Bounds in the Supplementary Material, Appendix. While our experimental sample is balanced on observable characteristics, simulated dropout rates in the video or joint treatment groups are much higher than in the test or control group.^b^ Our results are robust to estimating treatment effects under imputation. Lee Bounds are relatively tight for treatment effects of the personality test but are wide for treatment effects of the video and joint intervention groups. Combining the insights from our initial findings with results from our imputation and bounding exercises, we lack sufficient evidence to assert that the videos have a causal effect. Therefore, our results should be interpreted as suggestive.

Our suggestive results on the impact of video interventions are consistent with recent research on interventions targeting political polarization. For example, Voelkel et al. (13) show that the most effective among 25 interventions to reduce political animosity and correct democratic misperception was a video depicting a series of interactions between pairs of people with different political orientations, but that are able to connect and find mutual respect. Our paper contributes to the existing literature that has attempted to understand how emotions and reason affect vulnerability to fake news (14–17) by providing experimental evidence on debiasing interventions from an under-studied context. Among the papers in (18) out of 81 studies, none took place in Colombia. Importantly, our study provides evidence on the effectiveness of debiasing interventions that do not teach media literacy skills to detect misinformation directly. In contrast, our interventions aim at nudging critical thinking skills that allow individuals to detect their own biases which in turn make them more vulnerable to misinformation. Our interventions are, in spirit, similar to prebunking and inoculation interventions as those studied in (19–21) where subjects learn about misinformation techniques. In this sense, our video interventions are most similar to (22). Nudging critical thinking skills that encourage individuals to draw conclusions from their own experiences and available data, however, can have important implications for the economics of misinformation and polarization in environments that rapidly employ new technologies.

We view our study as containing three key insights on the demand side. First, videos that provoke viewers to slow down and reconsider their thinking are correlated with higher skepticism and less misinformation vulnerability. Yet, our assessment that aims at a similar result by providing individuals with evidence about their cognitive traits has limited effects. Second, the joint intervention could potentially affect the spread of misinformation in that video-and-test-treated individuals seem more likely to report a tweet as misinformation. This provides some hope that demand side factors can have spillover effects to the supply side of this market. Third, we find suggestive evidence that the videos encourage viewers to dehumanize less and to reflect more critically on their discrimination experiences.

## Context and experimental design

### Colombian presidential election in 2022

At the beginning of 2022, the retiring right-wing president of Colombia, Ivan Duque, had only 25% of the populations’ approval. Results of the primaries in March showed that the center lacked support and that citizens were seeking radical change. After the primaries two opposite candidates emerged as favorites, polarizing the country: Rodolfo Hernandez, claiming to be independent but supported by the right-wing parties, and Gustavo Petro, a left-wing senator and former guerrilla member.

This polarized environment was perfect for misinformation to arise. Journalists in the region compared the 2022 elections to “a schoolyard food fight, noticing multiple trends in mis—and disinformation being slung from left, right and center.” Fact checking outlets, like *Colombiacheck* saw an increase in new outlets spreading misinformation and a high volume of decontextualized photos of candidates regardless of their political orientation.

### Experimental design

In this context, Ethos Behavioral Team (Ethos BT) created the ^c^ DIP a digital and information literacy project based in behavioral science. Its purpose is to provide tools to help people and organizations to be more critical of information. We partner with Ethos BT to investigate the efficacy of various tools aimed at bolstering critical thinking skills to mitigate vulnerability to misinformation. Following (7), our focus lies predominantly on interventions designed to fortify one of critical thinking’s dual pillars: developing and assimilating empirical evidence and updating one’s beliefs. To do so, we run an online framed field experiment (23) that explores three different tools: a thought-provoking video that shows how behavioral biases can affect our everyday lives, a personality test that shows how prone we are to automatic thinking and misperceptions, and a combination of the video and the personality test. This study was reviewed by the University of Chicago IRB (IRB22-1217).^d^

Furthermore, because we wish to test theory and speak to policymakers, we use “Option C thinking” (24). To do so, we examine whether four psychological traits identified by DIP as correlates of vulnerability to misinformation are mediators of the relationship between our interventions targeting critical thinking enhancement and our outcome of misinformation vulnerability. (25) argues that psychological risks, including political polarization, and the intertwining of emotionality and morality, are key factors affecting the path between the exposure to and sharing of misinformation. Based on this, DIP focused on identifying these psychological risks to design the interventions we evaluate in this paper. DIP ran a prior survey experiment with 1,038 workers from five Colombian companies to identify psychological risks related to misinformation vulnerability. With these data, DIP identified four psychological traits associated with misinformation vulnerability: trust, dehumanization, discrimination perception, and ambiguity aversion.

Participants were randomized into one of three treatment groups or a control group without intervention. Those who were in the video only group were randomly exposed to one of four distinct videos that follow the same structure, but focus on a different mediator. The videos show interactions between real people^e^ in a neutral setting. Each interaction brings together people from distinct social groups in Colombia. The first part of the video introduces each person to the viewer and shows how the participants get to know each other free of any identity attribution. In this interaction, they share personal stories that visibly sensitize the respective other(s). The videos then show sequences that were filmed before the interaction. In those sequences, each person makes statements about the other social group(s)—based on societal stereotypes and stigmas. The video shows them watching these sequences together with their counterpart(s) and then reflecting on their former statements, revising them, and acknowledging that they made fast statements based on stereotypes. The videos encourage the viewer to question their own cognitive traits by “slowing down” their thinking.

Like the videos, there are four personality tests that follow the same structure, but each focuses on a different potential mediator. In each test, participants are asked to answer questions about themselves and their political preferences. Then, they are exposed to a set of statements that depend on the mediator we are testing. After completing the survey, respondents receive their test results. The results explain what the test is measuring and the respondent’s biases and vulnerability to misinformation in terms of the four mediators.

With three treatments (D=3) and four potential mediators (M=4), we adopt a *partial* factorial design randomization. In total, we have D×M=12 treatment groups and one control group. Due to logistical constraints, we did not implement a full factorial design (16 treatments groups) including the interaction of all the mediators with the control group, meaning that the control group only responded to the survey but did not receive any placebo interventions.

### Data

To elicit misinformation vulnerability, participants are presented with 19 distinct news headlines in random order and are prompted to determine their reliability as in (21). These headlines are curated from articles reviewed by fact-checking platforms such as *ColombiaCheck* or *El Detector*, affiliated with the national news outlet *La Silla Vacía*, ensuring the reliability of their truthfulness. Additionally, we categorize the news articles based on their political ideology to explore potential correlations between susceptibility to misinformation and political inclinations. Among the 19 selected headlines, ten are fake and nine are authentic, or actually true, news. Of the fake headlines, four lean towards the left, four lean towards the right, and two are neutral. Conversely, among the true headlines, three lean towards the left, three lean towards the right, and three are neutral. All statements, whether true or false, are polarizing in the sense that it is not clear if they are true or false and would probably need to pass through a fact-checker to confirm their veracity. The true news therefore share characteristics with fake news. News headlines were presented next to logos imitating news agencies (neither names nor logos actually exist in Colombia) as in (21). This was to prompt participants to think about information coming from news outlets rather than social networks though to avoid deception we did not explicitly link any headlines with logos.

Following the classification of the news headlines, participants are presented with two tweets and asked to assess their appropriateness and indicate if they would report them. Both tweets are fictitious, with one (Tweet A) devoid of political content, whereas the other (Tweet B) carries political connotations. Lastly, participants are prompted to respond to questions concerning our four potential mediators. Upon completing the survey, all participants receive a bonus to purchase an ice cream from a renowned ice cream shop with branches across the country. We acknowledge that a limitation of this study is that the outcomes are measured immediately after treatment. In future research, we aim to assess whether our treatments have persistent effects over time.

The intervention was made using *Datasketch*, a Colombian firm similar to Qualtrics. Unfortunately, we were unable to track completion rates as *Datasketch* only retained data from participants who completed the entire survey. Nonetheless, in Supplementary Material, Appendix section Attrition, we perform two statistical exercises: imputation and bounding the average treatment effects as in (26), to address concerns about potential differential attrition. Despite challenges with attrition, we can confidently affirm that those who completed the survey complied to their assigned treatment, as they were unable to fast-forward the video or skip questions in the personality test.

In total, 3,297 individuals completed our online framed field experiment. However, our final sample is composed of 2,235 individuals after eliminating duplicates based on identifiers built upon national identification numbers and surnames. One reason we might observe the same individual appearing multiple times in the survey is that participants could share their unique links with family or friends. This results in multiple entries appearing as if they are from the same person in our dataset. To ensure we retain data from the intended respondents, we only keep the first observation for each individual. Of those remaining, 645 individuals are in the control group, 750 are treated with the personality test only, 408 are treated with the video only, and 432 are treated with both the personality test and the video.

The mean age in our sample is 33.5 years and all subjects are eligible to vote in elections (over 18 years old). The sample is balanced in terms of gender, with 52.8% of the sample being women. In terms of education level, 37.8% have some level of school education, 30.1% have a technical degree, 16.1% have a professional degree, and 9.4% have a graduate degree. In terms of place of residence, 58.1% of the sample is living in Bogota, and the rest live in other regions of the country.

### Sampling strategy

We compose our sample by inviting individuals from the general Colombian population to participate in our experiment. The invitation mentioned that they would participate in a survey and could win a small prize. We attempted to be as generic in our call as possible to avoid experimenter demand effects. We pursued three primary dissemination channels: (i) Leveraging social networks such as Facebook, LinkedIn, Twitter, and WhatsApp to reach a broad audience. (ii) Collaborating with a prominent journalist and TV presenter renowned for their extensive and diverse following across Colombia. (iii) Partnering with private enterprises to distribute the intervention among their employees, enhancing its reach and impact.

Our underlying population of interest is the Colombian population that has access to digital platforms and social media. Our sample predominantly consists of individuals residing in Bogotá, the capital city of Colombia. While we recognize the importance of obtaining a more diverse representation from across the country, insights gathered from Bogotá residents are still highly informative for addressing our research question. As of January 2022, Bogotá accounted for approximately 10–12% of the nation’s total population, with around 30% of its residents being from outside the city. Additionally, given Colombia’s centralized political system, many pivotal decisions are made in Bogotá, further underscoring the significance of understanding the behaviors and attitudes of its inhabitants.

## Empirical strategy

Our general empirical approach will be standard and follows the typical analysis of data when the assignment mechanism is controlled. Let $Y_{i}$ for unit *i* be our outcome of interest, let $D_{i}=\{0,1,2,3\}$ be our treatment variable, where $D_{i}=0$ is the control group, $D_{i}=1$ is the personality test only, $D_{i}=2$ is the video only and $D_{i}=3$ is the treatment with both the personality test and the video. We group the distinct videos and personality tests together into one category each for this analysis and do not distinguish between the different types of videos or personality tests.

Let $M_{i}=\{0,1,2,3\}$ be our mediator variable, where $M_{i}=1$ is ambiguity aversion, $M_{i}=2$ is dehumanization, $M_{i}=3$ is perceived discrimination and $M_{i}=4$ is trust. Our treatments $D_{i}$ can yield an effect directly on $Y_{i}$, but also they can yield an effect indirectly on $Y_{i}$ through the direct effect of $D_{i}$ on $M_{i}$, holding the direct contribution of $D_{i}$ fixed.

Accordingly, we define the ATE as:


$$\tau =E[Y_{i}(M_{i}(d),d),-Y_{i}(M_{i}(0),0)].$$

Assuming a parametric functional form, we estimate the following regression:


(1) $$Y_{i}=\beta_{0}+\beta_{1}D_{i1}+\beta_{2}D_{i2}+\beta_{3}D_{i3}+X_{i}^{\prime }\gamma +\mu_{i}$$

where $D_{id}$ is equivalent to $D_{i}=d$ and $X_{i}$ is a vector of control variables including age, gender, education level and whether the subject is from Bogota or anywhere else in Colombia. We are interested in $\beta_{1}$, $\beta_{2}$, and $\beta_{3}$.

Since towards the end of our intervention we asked participants questions related to our four potential mediators, with our data we can also speak to the effect of $D_{i}$ on $M_{i}$. In particular, because our treatment $D_{i}$ is statistically independent of potential outcomes $Y_{i}(M_{i}(d),d)$ and potential mediators $M_{i}(d)$, we can estimate:


(2) $$M_{i}=\alpha_{0}+\alpha_{1}D_{i1}+\alpha_{2}D_{i2}+\alpha_{3}D_{i3}+X_{i}^{\prime }\delta +\nu_{i}$$

for each of our four mediators of interest. We are interested in the coefficients $\alpha_{1},\alpha_{2}$, and $\alpha_{3}$. $X_{i}$ is the same vector of controls we use in Eq. 1.

## Results

We summarize our results in two sections. First, we present the main treatment effect estimates. This summary includes direct estimates on our outcome metrics. In addition, we explore how our treatments affect the supply side of misinformation via the report of fake news. Then, in section 4.2 we consider potential mechanisms more carefully.

### Main results

Our results suggest that the video intervention reduces the likelihood of participants deeming fake headlines as reliable. In Table 1, we show that results from estimating Eq. 1 reveal that those exposed to the video were 30.3% (4 pp) less likely to view any fake news as trustworthy compared to the control group (column (1)). This effect is even more pronounced when considering neutral headlines, with individuals being 32.3% (7.4 pp) less likely to perceive neutral fake news as credible compared to a control mean of 23% (column (2)). Effects for political headlines are similarly large, decreasing the likelihood that news is considered reliable by 5.3pp compared to a control mean of 17.4% for right-leaning news and a control mean of 20.6% for left-leaning news.

**Table 1.** News.

	Fake Headlines				True Headlines			
	(1)	(2)	(3)	(4)	(5)	(6)	(7)	(8)
	All	Neutral	Right	Left	All	Neutral	Right	Left
Test	0.014	−0.000	0.023	−0.019	0.014	0.011	−0.016	0.011
	(0.018)	(0.022)	(0.020)	(0.021)	(0.019)	(0.021)	(0.022)	(0.023)
Video	−$0.040^{b}$	−$0.074^{c}$	−$0.053^{b}$	−$0.053^{b}$	−0.020	−$0.051^{b}$	−0.042	0.005
	(0.019)	(0.024)	(0.021)	(0.023)	(0.021)	(0.023)	(0.025)	(0.026)
Both	−$0.039^{b}$	−$0.065^{c}$	−$0.039^{a}$	−$0.062^{c}$	−$0.044^{b}$	−$0.042^{a}$	−$0.073^{c}$	−0.028
	(0.018)	(0.024)	(0.021)	(0.023)	(0.019)	(0.022)	(0.024)	(0.025)
*P*-value Test = Video	0.003	0.001	0.000	0.116	0.088	0.005	0.286	0.824
*P*-value Video = Both	0.968	0.699	0.539	0.716	0.239	0.704	0.222	0.238
Control Mean	0.132	0.229	0.174	0.206	0.143	0.195	0.236	0.226
Observations	2235	2235	2235	2235	2235	2235	2235	2235
R-Squared	0.064	0.060	0.059	0.048	0.061	0.063	0.033	0.017

SEs in parentheses.

^*a*^ $P<0.10{,}^{b}P<0.05{,}^{c}P<0.01$ .

The personality test alone neither yielded a significant impact on participants’ perceptions of the truthfulness of fake headlines, nor did it provide a synergistic benefit when combined with the video intervention. Although the joint treatment did reduce vulnerability to misinformation, its effectiveness is slightly smaller compared to the video intervention for right-leaning and neutral news.

Notably, our video intervention is also effective in decreasing participants’ likelihood to perceive neutral true headlines as reliable. It does not have statistically significant impacts on true political news reliability, however.

Further, the joint intervention (personality test and video) seems to be effective in decreasing participants’ likelihood to perceive overall true headlines as reliable, with the exception of left-leaning headlines. Those who received the joint intervention are 30.7% (4.4 percentage points) less likely to regard true headlines as trustworthy. This outcome underscores how our video intervention when combined with the personality assessment fosters critical thinking by instilling skepticism toward all headlines, yet it falls short in equipping individuals with the discernment to differentiate between authentic and fabricated information.

In Table 2, columns (1) and (3) assess participants’ perception of the appropriateness of tweet A and tweet B, respectively. In columns (2) and (4), the focus shifts to participants’ willingness to report tweet A and tweet B, respectively. Recall that both tweets are fake. Tweet A is nonpolitical and states “Vaccine fraud is being uncovered. With more affected people doubly injected every day, when fall arrives panic will reign.” Tweet B is political, noting “The President is writing what would be a fragment of the state of “internal commotion” throughout the national territory.”

**Table 2.** Tweets.

	Tweet A		Tweet B	
	(1)	(2)	(3)	(4)
	Inappropriate	Report	Inappropriate	Report
Test	−0.021	0.022	−0.020	0.002
	(0.024)	(0.026)	(0.024)	(0.027)
Video	0.025	0.044	−0.014	0.030
	(0.027)	(0.031)	(0.028)	(0.031)
Both	−0.005	$0.085^{c}$	0.020	0.037
	(0.027)	(0.030)	(0.027)	(0.031)
*P*-value Test = Video	0.081	0.470	0.812	0.351
*P*-value Video = Both	0.301	0.204	0.257	0.823
Control Mean	0.735	0.584	0.709	0.571
Observations	2235	2235	2235	2235
R-Squared	0.047	0.011	0.062	0.009

SEs in parentheses.

^*a*^ $P<0.10{,}^{b}P<0.05{,}^{c}P<0.01$ .

We find no statistically significant effect of any of our interventions increasing the percentage of people who consider the tweets as inappropriate. We attribute this result to the fact that the percentage of individuals in the control group who considered that the tweets were inappropriate was already very high (over 70% for both tweets), making the effect of the treatments small in magnitude and statistically insignificant. Yet, our findings do indicate that individuals exposed to the joint intervention (video and behavioral assessment) are 15% (8.5 percentage points) more inclined to express willingness to report Tweet A as fake. This result suggests that our intervention exerts some influence on reporting nonpolitical fake news, while exhibiting no impact on reporting fake political news. This result should be more carefully explored in future work as it highlights that a demand side intervention can potentially have spillover effects to the supply side, or the spread of disinformation.

Since differential attrition is a concern when analyzing the first set of results, in Supplementary Material, Appendix section Attrition we perform two statistical exercises. We first do an imputation exercise, and then we construct bounds on average treatment effects following the approach of (26). Results from the imputation exercise are reported in Supplementary Material, Appendix, Table A2. In comparison to the original results presented in Table 1, only minor changes in the level and significance of the coefficients can be observed. The main results, however, remain consistent with the original. The results from the bounding exercise are in Table A3 for the test treatment effects, in Table A4 for the video treatment effects, and in Table A5 for the joint-intervention treatment effects. We observe that for the test treatment effects, the bounds are tight and do not contain the zero, validating our original findings. However, for the video and the joint-intervention treatment effects, the bounds are too wide to be informative of the true treatment effect. Based on our initial findings, along with the results from our imputation and bounding exercises, we can conclude that our results are suggestive rather than definitive. We lack sufficient evidence to assert that the videos have the desired causal effect.

### Potential mechanisms

Results from estimating Eq. 2 are reported in Table 3. Column (1) examines whether participants perceive discrimination based on income, race, gender, or social strata in their daily lives. Column (2), examines whether participants feel discomfort with uncertain situations (ambiguity aversion). Column (3) examines whether participants trust others in general. Lastly, Columns (4) to (6) gauge whether participants report negative emotions, such as recklessness, shame, or suspicion, towards voters of specific political views.

**Table 3.** Discrimination, ambiguity and trust.

	Discrimination (1)	Ambiguity Aversion (2)	Trust (3)	Dehumanization		
				Left	Neutral	Right
				(4)	(5)	(6)
Test	0.014	0.017	−0.006	−0.026	0.009	−0.003
	(0.022)	(0.018)	(0.026)	(0.025)	(0.025)	(0.024)
Video	0.036	0.020	0.024	−$0.097^{c}$	−0.011	−$0.058^{b}$
	(0.027)	(0.021)	(0.031)	(0.031)	(0.029)	(0.030)
Both	0.028	0.032	0.020	−$0.078^{c}$	−0.031	−$0.056^{a}$
	(0.026)	(0.022)	(0.030)	(0.030)	(0.029)	(0.029)
*P*-value Test = Video	0.401	0.864	0.309	0.019	0.483	0.055
*P*-value Video = Both	0.775	0.632	0.895	0.589	0.509	0.938
Control Mean	0.231	0.122	0.597	0.674	0.676	0.693
Observations	2,235	2,235	2,235	2,235	2,235	2,235
R-Squared	0.083	0.010	0.021	0.029	0.069	0.041

SEs in parentheses.

^*a*^ $P<0.10{,}^{b}P<0.05{,}^{c}P<0.01$ .

Our analysis reveals no significant impact on discrimination, trust towards others, or ambiguity aversion from any of our interventions excluding those factors as mediators in our experiment. However, watching the video leads to a decrease in the use of negative emotions to characterize both the political left and right by 14% (9.7 percentage points) and 8% (5.8 percentage points), respectively, suggesting a reduction in dehumanization.

The personality tests alone show no significant effect on any of the four potential mediators studied, and when combined with the video intervention, they provide no additional benefit. Overall, therefore, we find limited evidence that the proposed mediators in the literature have some power as a causal pathway. We view this result as merely a first step in understanding the complete relationship between potential interventions and key outcome metrics around misinformation, but a promising one.

## Conclusion

The causes and consequences of misinformation has become a key concern across governments, organizations, and scientists. For example, whether misinformation spreads like wildfire or dies quickly on the vine merits serious consideration. And, understanding the factors that moderate that spread is invaluable. In this study, we conduct an online framed field experiment that focuses on the demand side of misinformation. In particular, we focus on enhancing critical thinking skills in an effort to alter the demand side in this market.

Our work yields several insights. First, our video intervention seems to affect the level of individual skepticism: whether fake or real, our subjects are more skeptical of any news. We argue that this is socially optimal given the polarizing nature of both the true and fake news that we presented because of the characteristics of the true news that we shared. Given their similarity in tone to the fake news that we shared, this encourages subjects to investigate truthfulness further. Second, we find suggestive evidence that our intervention affects the spread of misinformation in that individuals treated with both the video and the personality test are more likely to report a tweet as misinformation. This provides a silver lining in that reducing the demand of fake news could deliver on the dual goal of reducing the spread of fake news by encouraging reporting of misinformation. Third, we find interesting mediation paths, as we report suggestive evidence that the videos seem to encourage viewers to dehumanize less and to reflect more critically on their discrimination experiences.

In closing, we would be remiss not to mention the generalizability of our results and future research directions. In terms of the former, we follow (27) SANS conditions to understand the generalizability of these results. Our sample is a selected subset of Colombian individuals who agreed to participate in an online experiment. In this sense, whether the results generalize to other Colombian’s, much less non-Colombian citizens is an open question. In terms of attrition, we try to address concerns by performing statistical methods like imputation and bounds as robustness checks. Considering the naturalness of the choice task, setting, and time frame, we use an online framed field experiment; thus, our setting is one in which individuals are engaged in a natural task and margin. Finally, in terms of scaling our insights, the interventions are simple and easy to administer so we argue that the benefit–cost profile should be enhanced at scale as the program expands. Finally, since we view our demand side results as WAVE 1 (Efficacy and Proof of Concept) insights, in the nomenclature of (27), replications need to be completed to understand if our results can be applied to other populations.

This point naturally leads to future research directions. Beyond replications, we urge researchers to more fully explore the suite demand side interventions in this market. This naturally begins with experimenting with several types of approaches, from pecuniary to nonpecuniary interventions, as well as the moderators and mediators of those effects. In this spirit, we trust that scholars will engage in creating causal moderators rather than stop at merely descriptive variants. Such explorations will help to detail what works and why. In addition, we trust that future work will tackle the interplay of the demand and supply sides of the misinformation market. Until key features are understood about the production and consumption sides, and relevant interactions, efficient policies to combat the creation and spread of misinformation will be difficult, if not impossible.

## Supplementary Material

pgae361_Supplementary_Data

## Data Availability

The data underlying this article are available in the repository “Critical Thinking and Misinformation” at https://github.com/linaramirez0604/critical-thinking-misinformation, and is publicly available.

## Notes

a These mediators have been found to be potential drivers of misinformation vulnerability in a polarized context such as Colombia in a prior survey experiment conducted by Detox Information Project (DIP).

b We cannot test for dropout rates directly due to data restrictions but use data from a subsequent data round and randomization simulations to calculate dropout rates for all groups. Although higher dropout rates in both video groups suggest that those might be driven by technical difficulties and phone data limitations, we cannot exclude that dropout might be driven by unobservables that are linked to misinformation vulnerability and news skepticism.

c See the project here: https://www.somosdip.com/.

d Informed consent was waived because this study involves minimum risk.

e The protagonists of the videos were not actors. The videos can be found under the following links: Stereotyping/ambiguity video: https://www.youtube.com/watch?v=eKbb5RE5cw0; Discrimination video: https://www.youtube.com/watch?v=wt8-XD5AAls; Trust video: https://www.youtube.com/watch?v=oeK1lN4pkbI; Dehumanization video: https://www.youtube.com/watch?v=2TzbFrJFoxQ.

## Previous Presentation

These results were previously presented at SEEDEC, August, 2022; Political Economics Workshop at Universidad del Rosario, November, 2022; USAID, November, 2022; LACEA, November, 2023; Polarization Workshop at MIT November, 2023.

## Preprints

A preprint of this article is published at DOI: 10.3386/w32367.
