# Supplementary material for: Critical thinking and misinformation vulnerability: Experimental evidence from Colombia
Source: PNAS Nexus. 2024 Oct 15;3(10):pgae361. doi: 10.1093/pnasnexus/pgae361 (PMC11475746; doi:10.1093/pnasnexus/pgae361)
Supplement: pgae361_Supplementary_Data [file pgae361_supplementary_data.zip › supplementary-material.pdf]

PNAS Nexus, 2024, pp. 1–4

doi: DOI HERE
Advance Access Publication Date: 30/05/2024
Paper

Draft

PAPER

Critical Thinking and Misinformation Vulnerability: Experimental Evidence from Colombia

John A. List,[a] Lina M. Ramirez,[a,*] Julia Seither,[b] Jaime Unda[c] and Beatriz Vallejo[c]

[a]Department of Economics, University of Chicago, Chicago, Illinois, United States, [b]Department of Economics, Universidad del Rosario, Bogota, Cundinamarca, Colombia and [c]Ethos Behavioral Team, Bogota, Cundinamarca, Colombia
[*]Corresponding Author: lmramirez@uchicago.edu.
FOR PUBLISHER ONLY Received on Date Month Year; accepted on Date Month Year

Abstract

Misinformation represents a vital threat to the societal fabric of modern economies. While skills interventions to detect misinformation such as de-bunking and pre-bunking, media literacy, and manipulation resilience have begun to receive increased attention, evidence on de-biasing interventions and their link with misinformation vulnerability is scarce. We explore the demand for misinformation through the lens of augmenting critical thinking in an online framed field experiment during the 2022 Presidential election in Colombia. Data from roughly 2.000 individuals suggest that providing individuals with information about their own biases (obtained through a personality test) has no impact on skepticism towards news. But (additionally) showing participants a de-biasing video seems to enhance critical thinking, causing subjects to more carefully consider the truthfulness of potential misinformation.

Key words: Fake News, Misinformation, Critical Thinking

Appendix

Attrition

Inferring attrition from the randomization strategy

Unfortunately, we encountered some challenges with the data collection process, which we disclose fully here and address to the best of our abilities. Our experiment was initially designed to include three rounds of data collection to assess the persistence of our treatments over time. The second and third wave were launched 1 and 2 months after the first wave, respectively. However, we were unable to utilize data from the second and third waves for two main reasons: i) Only about 1% of the participants in rounds two and three also participated in round one, which precluded our ability to measure persistence. ii) The company responsible for data collection, *Datasketch*, did not collect the

© The Author 2024. Published by Oxford University Press. All rights reserved. For permissions, please e-mail: journals.permissions@oup.com

Draft

treatment status for rounds two and three. Consequently, even if we wanted to use these waves independently, we could not.

Additionally, for the first wave, *Datasketch* retained data only from participants who completed the entire survey. It was not until the third wave that they began retaining data from participants who did not complete the survey.

In wave 3, we observed an attrition rate of 20%. Regrettably, because the treatment indicator was not collected in wave 3, we cannot determine if this attrition rate differs by treatment group. However, we leverage this attrition rate from wave 3 to infer the attrition rate by treatment in wave 1, based on the randomization procedure implemented by *Datasketch*.

We designed the experiment to randomize participants into one of three treatment groups or a control group. For those assigned to the treatment groups, participants were further randomized to watch one of four distinct videos, each focusing on a different mediator but following the same structure. However, the actual randomization strategy implemented was slightly different: participants were first randomized into one of the four mediator groups (dehumanization, discrimination, trust, or ambiguity) or a control group. Those in any of the mediator groups were then randomly assigned to one of the three treatments (video only, test only, or both).

We use the randomization strategy and the attrition rate of wave 3 to infer the attrition rate by treatment in wave 1. Our cleaned sample consists of 2,237 observations. To be conservative, we applied an attrition rate higher than the 20% observed in wave 3. We chose an attrition rate of 32%, leading to a desired sample size of 3,300. We conducted 1,000 simulations of the randomization procedure and calculated the mean number of observations per treatment group. In Table A1 we present the results. Column 1 shows the mean number of observations per treatment group that we obtain from simulating 1,000 times the randomization with a sample size of 3,300. Column 2 shows the actual number of observations that we have per treatment group. Column 3 calculates the probability of response given the simulated sample size. Column 4 reports how many observations are missing per treatment group. We can notice that according to this exercise the control and test group have small attrition, whereas the video and both treatments have quite large attrition. This makes sense because the control survey was the smallest of all, followed by the test one, and the video and the both were longer in magnitude.

Table A1. Simulated Response Rate

	$\mathbb{E}(N)$ (1)	N (2)	$\mathbb{P}(R_i)$ (3)	Missing (4)
Control	660	645	0.98	15
Test	880	752	0.85	128
Video	880	408	0.46	472
Both	880	432	0.49	448
Total	3300	2237		1063

Given these attrition rates, in the next sections we implement several statistical approaches to deal with attrition.

Imputation

Imputation is a commonly used approach if a dataset has missing observations. It allows for the missing data to be replaced with alternatively generated data. The underlying assumption behind this method is that attrition can be entirely explained by observable characteristics. Here, we are using mean value imputation method for the covariates and stochastic regression imputation for the outcomes to account for attrition in our sample.

To do the imputation, we first need to add the missing observations to our sample. As described in the previous subsection, we can infer the original number of observations in the absence of attrition from the randomization strategy. As shown in Table A1 our sample should have had 1,063 additional observations across the three treatment groups and the control group. Thus, we added 1,063 additional observations to our main dataset.

Now, to impute the value of the covariates we use the basic mean value imputation method since is the only one that fits our data limitations. We have access to data from the third round of the experiment in which we recorded

whether participants completed the experiment or exited prematurely. In this round, we first check if the mean of the covariates that we measure is statistically different depending on whether the survey was completed or not. Let X_i be our covariate of interest and $R_i \in \{0,1\}$ be an indicator of participation in the experiment. We calculate:

$$\mathbb{E}[X_i(R_i = 1) - X_i(R_i = 0)]$$

On average, we find that participants who are younger, male, do not live in Bogota, and have some degree of college education are less likely to complete the online experiment. Assuming that the subset of participants, who did not complete the experiment, is similar for round 1 and round 3, we compute the mean of the covariates for the sub-sample that attrited in the third round and impute them to our new observations in the main dataset. [1]

In order to impute the missing outcome variables, we use a stochastic regression imputation approach. We first re-estimate Equation (1) in the main paper:

$$Y_i = \beta_0 + \beta_1 D_{i1} + \beta_2 D_{i2} + \beta_3 D_{i3} + X_i'\gamma + \mu_i \quad (1)$$

where Y_i are the outcomes of interest, D_{id} is equivalent to $D_i = d$ and X_i is the vector of covariates. From the fitted regression model, we predict values for the missing observations. Additionally, we calculate the residuals and their standard deviation, and add this error term to the predicted values, and we replace the missing outcomes with the stochastically imputed values.

This method enables us to re-run Table 1 of the paper with an extended dataset that has imputed values for individuals who dropped out of the experiment. Table A2 includes the result of this imputation exercise. In comparison to the original results, only minor changes in the level and significance of the coefficients can be observed. The main results, however, remain consistent with original Table 1.

[1] As described in the previous subsection, we do not observe treatment statuses in the third round due to data limitations that prevent us from matching participants across waves. Thus, a more detailed breakdown of attrition in each treatment and the control group is not possible.

Lee Bounds

Adapting [1] and [2] to our context, let Z_i be the randomly assigned treatment. Let $Z_i = 1$ be the treatment (i.e test, video, or both), and let $Z_i = 0$ be the control. Let $R_i \in \{0,1\}$ be an indicator of participation in our experiment. Let Y_i be the outcome of interest. We are able to observe $(Y_i R_i, R_i, Z_i)$. $R_i(z)$ is the potential participation under treatment z. Likewise, $Y_i(z)$ is the potential outcome under treatment z. Our assumptions are i) Independence: $\{Y_i(z), R_i(z)\}_{z\in\{0,1\}} \perp Z_i$, and ii) Monotonicity: $P(R_i(0) \geq R_i(1)) = 1$. Notice that our monotonicity assumption establishes that the probability of response under the control group is greater or equal to the probability of response under any treatment. This assumption is reasonable in our context because it is more likely that participants in the control group will complete the experiment compared to those in the treatment groups, as the control condition is shorter in duration.

We are going to construct the bounds on individuals who participated under the treatment assignment:

$$\begin{aligned} \mathbb{E}[Y_i(1) - Y_i(0) | R_i(1) = 1] = \\ \underbrace{\mathbb{P}(Y_i(1) = 1 | R_i(1) = 1)}_{\text{Identified}} - \underbrace{\mathbb{P}(Y_i(0) = 1 | R_i(1) = 1)}_{\text{Unidentified}} \end{aligned} \quad (2)$$

The first term of Equation 2 is identified and equal to $\mathbb{P}(Y_i = 1 | Z_i = 1, R_i = 1)$, so we only need to bound the second term. Let's define $p_k = \mathbb{P}(R_i(1) = k | R_i(0) = 1)$. Notice that p_1 and p_0 are identified because $p_1 = \frac{\mathbb{P}(R_i(1)=1)}{\mathbb{P}(R_i(0)=1)}$ and $p_0 = 1 - p_1$. We have:

$$\begin{aligned} &\mathbb{P}(Y_i(0) = 1 | R_i(0) = 1) \\ &= \mathbb{P}(Y_i(0) = 1 | R_i(0) = 1, R_i(1) = 1) p_1 \\ &+ \mathbb{P}(Y_i(0) = 1 | R_i(0) = 1, R_i(1) = 0) p_0 \end{aligned}$$

Draft

Table A2. News - Imputation

	(1)<br>All Fake	(2)<br>Neutral Fake	(3)<br>Right Fake	(4)<br>Left Fake	(5)<br>All True	(6)<br>Neutral True	(7)<br>Right True	(8)<br>Left True
Test	0.019	-0.001	0.022	-0.022	0.013	0.006	-0.025	0.016
	(0.017)	(0.021)	(0.019)	(0.020)	(0.018)	(0.020)	(0.021)	(0.022)
Video	-0.045***	-0.078***	-0.052***	-0.055***	-0.012	-0.044**	-0.048**	0.000
	(0.017)	(0.021)	(0.019)	(0.020)	(0.018)	(0.020)	(0.022)	(0.022)
Both	-0.034**	-0.062***	-0.052***	-0.071***	-0.044***	-0.050**	-0.079***	-0.032
	(0.017)	(0.021)	(0.019)	(0.020)	(0.017)	(0.020)	(0.021)	(0.022)
p-value Test = Video	0.000	0.000	0.000	0.069	0.134	0.006	0.226	0.442
p-value Video = Both	0.466	0.369	0.982	0.349	0.030	0.693	0.091	0.099
Control Mean	0.134	0.230	0.176	0.208	0.142	0.198	0.239	0.227
Observations	3298	3298	3298	3298	3298	3298	3298	3298
R-Squared	0.049	0.042	0.043	0.034	0.044	0.045	0.023	0.012

Standard errors in parentheses
* $p < 0.10$, ** $p < 0.05$, *** $p < 0.01$

Draft

Re-arranging this equation we get:

$$\begin{aligned}\mathbb{P}(Y_i(0) = 1 | R_i(0) = 1, R_i(1) = 1) \\ = \frac{1}{p1}\,\mathbb{P}(Y_i(0) = 1 | R_i(0) = 1) \\ -\frac{p_0}{p_1}\,\mathbb{P}(Y_i(0) = 1 | R_i(0) = 1, R_i(1) = 0)\end{aligned}$$

Notice that $\mathbb{P}(Y_i(0) = 1 | R_i(0) = 1) = \mathbb{P}(Y_i = 1 | Z_i = 0, R_i = 1)$ is identified. In addition, we know that $\mathbb{P}(Y_i(0) = 1 | R_i(0) = 1, R_i(1) = 0) \in [0, 1]$ so we can propose the following bounds:

$$\phi_{\text{lb}} = \max\left\{0, \frac{1}{p_1}\,\mathbb{P}(Y_i = 1 | Z_i = 0, R_i = 1) - \frac{p_0}{p_1}\right\}$$

$$\phi_{\text{ub}} = \min\left\{1, \frac{1}{p_1}\,\mathbb{P}(Y_i = 1 | Z_i = 0, R_i = 1)\right\}$$

And so we finally can get that $\mathbb{E}[Y_i(1) - Y_i(0) | R_i(1) = 1] \in [\Delta_{\text{lb}}, \Delta_{\text{ub}}]$:

$$\Delta_{\text{lb}} = \mathbb{P}(Y_i = 1 | Z_i = 1, R_i = 1) - \phi_{\text{ub}}$$

$$\Delta_{\text{ub}} = \mathbb{P}(Y_i = 1 | Z_i = 1, R_i = 1) - \phi_{\text{lb}}$$

Tables A3, A4, and A5 present the results of the bounding exercise for the test, video, and combined interventions, respectively. The first column shows the participant mean for each of the eight binary outcome variables considered in the main paper. The second and third columns provide the estimated lower and upper bounds for the effect of being assigned to the control group relative to the treatment group on these outcome variables.

Table A3 indicates that, when comparing the test group to the control group, the bounds contain zero only for true headlines leaning to the right and left (Right True and Left True). For the other outcomes, the bounds do not contain zero and are relatively tight, which aligns with our estimate of very low attrition in the test group.

In contrast, Tables A4 and A5 reveal that, when comparing the video group and the combined interventions group to the control group, all estimated bounds contain zero and are relatively wide. Despite our efforts, these wide bounds limit the informativeness of the results.

References

1. Deniz Dutz, Ingrid Huitfeldt, Santiago Lacouture, Magne Mogstad, Alexander Torgovitsky, and Winnie Van Dijk. Selection in surveys: Using randomized incentives to detect and account for nonresponse bias. Technical report, National Bureau of Economic Research, 2021.
2. David S Lee. Training, wages, and sample selection: Estimating sharp bounds on treatment effects. *Review of Economic Studies*, 76(3):1071–1102, 2009.

Draft

Table A3. Lee Bounds: Test vs. Control

Outcome	Mean	LB (se)	UB (se)
All Fake	0.044	0.028	0.147
		(0.016)	(0.013)
Neutral Fake	0.043	0.015	0.168
		(0.020)	(0.020)
Right Fake	0.054	0.033	0.185
		(0.018)	(0.018)
Left Fake	0.020	-0.006	0.147
		(0.018)	(0.018)
All True	0.040	0.022	0.158
		(0.017)	(0.014)
Neutral True	0.042	0.017	0.170
		(0.019)	(0.019)
Right True	0.023	-0.007	0.146
		(0.019)	(0.019)
Left True	0.023	-0.011	0.142
		(0.019)	(0.019)

Note: Standard errors of the estimated bounds are presented below the estimates and are computed using 500 bootstrap iterations.

Table A4. Lee Bounds: Video vs. Control

Outcome	Mean	LB (se)	UB (se)
All Fake	-0.046	-0.189	0.081
		(0.023)	(0.014)
Neutral Fake	-0.067	-0.306	0.145
		(0.026)	(0.017)
Right Fake	-0.062	-0.255	0.110
		(0.026)	(0.016)
Left Fake	-0.038	-0.244	0.144
		(0.026)	(0.018)
All True	-0.027	-0.181	0.109
		(0.023)	(0.015)
Neutral True	-0.056	-0.269	0.132
		(0.027)	(0.017)
Right True	-0.024	-0.262	0.187
		(0.029)	(0.020)
Left True	0.000	-0.254	0.225
		(0.030)	(0.020)

Note: Standard errors of the estimated bounds are presented below the estimates and are computed using 500 bootstrap iterations.

Table A5. Lee Bounds: Both vs. Control

Outcome	Mean	LB (se)	UB (se)
All Fake	-0.043	-0.170	0.084
		(0.020)	(0.014)
Neutral Fake	-0.053	-0.261	0.156
		(0.026)	(0.017)
Right Fake	-0.045	-0.215	0.124
		(0.024)	(0.016)
Left Fake	-0.050	-0.233	0.134
		(0.024)	(0.016)
All True	-0.052	-0.194	0.091
		(0.022)	(0.014)
Neutral True	-0.042	-0.228	0.144
		(0.026)	(0.017)
Right True	-0.061	-0.281	0.158
		(0.026)	(0.018)
Left True	-0.038	-0.269	0.193
		(0.028)	(0.018)

Note: Standard errors of the estimated bounds are presented below the estimates and are computed using 500 bootstrap iterations.
